# Mental health and educational support strategies for students with learning disabilities in Sub-Saharan Africa

**DOI:** 10.1097/MS9.0000000000005303

**Published:** 2026-07-14

**Authors:** Dominica Chisom Emelife, Victor Oluwafemi Femi-Lawal, Austed Mzuma, Bakare Abdulbasit Adedayo, Ebube Nwosu Clinton, Akinwale Olutobi James

**Affiliations:** a Mental Health Portfolio, Public Health en Afrique, Johannesburg, South Africa; b Malawi Epidemiology and Intervention Research Unit, Karonga, Malawi; c College of Medicine, University of Ibadan, Ibadan, Nigeria; d Faculty of Pharmaceutical Sciences, Chukwuemeka Odumegwu University, Anambra State, Nigeria; e Igbinedion University Okada, Edo State, Nigeria

**Keywords:** educational support, inclusive education, learning disabilities, mental health, students, Sub-Saharan Africa

## Abstract

Learning disabilities limit students’ participation in education and pose restrictions on their daily functioning, as they are often excluded from mainstream schools and lack adequate support due to stigmatization, poor inclusive teaching resources, and limited trained special educators. This narrative review aims to examine the relationship between mental health and educational support strategies for students with learning disabilities in Sub-Saharan Africa.

For this review, a literature search was conducted using databases including PubMed, Scopus, and Google Scholar, alongside organizational reports from the United Nations Children’s Fund, United Nations Educational, Scientific and Cultural Organization, and World Health Organization repositories. The search was narrowed down to studies published between 2010 and 2025, using search terms such as “learning disabilities,” “learning disorders,” “neurodevelopmental disorders,” “mental health,” “psychosocial,” “child development,” “education,” “school-based,” “inclusive education,” and “Sub-Saharan Africa,” as well as specific SSA country names. Due to the heterogeneous nature of the study designs, a narrative review was considered appropriate.

Results from the reviewed studies showed that students with learning disabilities experience common mental health challenges, particularly depression, anxiety, substance use, and social stigma. Identified support strategies include early childhood development centers offering assessment and therapy, the use of alternative communication methods, and caregiver training. However, implementation is hindered by a lack of mental health literacy, a shortage of professionally trained special educators, and the government’s inadequate focus on inclusive education.

Overall, the review highlights the need for multi-sectoral approaches to improving the educational and mental health outcomes of students with learning disabilities in Sub-Saharan Africa.

## Introduction

According to the World Health Organization (WHO), disability is a broad term that includes activity limitations, participation restrictions, and impairments^[^1^]^. It is viewed as an interplay between an individual’s bodily processes and the characteristics of their living environment, rather than solely a medical condition. Compared to other regions, Africa has a greater frequency of severe and moderate impairments^[^1^]^.

HIGHLIGHTS

- Mental health challenges among students with learning disabilities in Sub-Saharan Africa are underrecognized and underaddressed.
- Educational support systems play a critical role in shaping mental health outcomes for learners with disabilities.
- Current interventions are fragmented, with limited integration between the health and education sectors.
- Context-appropriate, school-based, and community-supported strategies show promise but remain insufficiently evaluated.
- Strengthening policy, teacher training, and cross-sector collaboration are essential for improving mental health and educational outcomes.

An estimated 240 million children worldwide, aged 0–17, have disabilities, with 28.9 million residing in Eastern and Southern Africa^[^2^]^. Only about one-third of these children attend primary school, and over half of them reside in rural areas^[^1^]^.

Many African nations are committed to advancing inclusive education, which entails meeting the needs of all students, including those with disabilities, in mainstream classrooms as opposed to placing marginalized children in separate special education classrooms^[^3^]^. However, a significant number of African children with disabilities continue to be excluded from school^[^4^]^. This is due to factors such as stigma within communities and schools, inaccessible infrastructure, and a lack of resources for inclusive teaching, including trained teachers^[^3,5^]^.

According to some reports, children with developmental disabilities, which are lifelong conditions marked by difficulties in social, communication, and/or cognitive skills, including intellectual disability, autism, and attention-deficit hyperactivity disorder (ADHD), which first appear in early childhood, experience higher levels of exclusion compared to other groups of children with disabilities^[^6,7^]^.

Early childhood education (ECE) offers the opportunity to hone developmental skills that lead to the maximum possible degree of personal independence^[^8^]^. During these early years of life, linguistic, cognitive, motor, and social interaction qualities evolve rapidly. Personalized education initiatives are, therefore, more likely to have the greatest impact on all students during this period. When children and young people are unable to reach their developmental potential, it limits their potential contributions and independence, leading to social and economic repercussions for families and communities^[^8–10^]^.

For children and adolescents with physical disabilities to participate effectively in inclusive educational environments, proper physical adjustments are required. Co-locating therapeutic support in schools will ensure access to care and enable children and adolescents living with disabilities to receive these services with minimal disruption to learning. However, in most South Asian and sub-Saharan African (SSA) schools, such factors are rarely implemented^[^8,11,12^]^.

Africa has a large youth population, and by empowering students with disabilities through relevant and contextually appropriate education, the continent can enhance economic outputs and improve the quality of life of its people^[^13^]^. The National Educational Goals Panel emphasizes that supportive communities, schools, and families are essential to a child’s preparedness for school. The five components of a child’s school preparation include their general knowledge, learning styles, cognitive abilities, linguistic development, social and emotional growth, and physical health and motor development^[^14^]^.

### Background

Students with learning disabilities encounter problems that extend beyond academic performance, which often affect their social and psychological well-being. Internationally, learning disabilities are characterized by a persistent difficulty in reading, writing, or mathematics that is not caused by intellectual disability, sensory impairment, or insufficient schooling^[^15,16^]^.

Mental health problems are common among children with learning disabilities. Mental health in this context includes internalizing disorders such as anxiety and depression, externalizing behaviors, and neurodevelopmental conditions such as autism that influence learning and adaptation. These include behavioral challenges such as conduct disorder and hyperactivity, as well as difficulties in attaining social relationships. These difficulties often interact with academic struggles, creating a cycle in which poor learning outcomes exacerbate psychosocial distress, while untreated mental health needs continue to act as barriers to educational progress^[^17–19^]^.

Academic support plans have been developed to alleviate these challenges by adapting teaching and learning environments to the needs of diverse learners. These include evidence-based methods such as differentiated instruction, remedial and small-group teaching, peer-assisted learning, teacher training in inclusive pedagogy, and the use of assistive technologies, alongside supportive policy frameworks^[^20^]^.

Focusing on SSA, the burden of disability among children remains high, but support systems are not well established. While most countries have made policy commitments to inclusive education, effective implementation is hindered by limited financing, inadequate teacher preparation, and weak monitoring structures. As a result, numerous children with learning disabilities lack access to proper academic and psychosocial support^[^16,21,22^]^.

Therefore, combining inclusive education with mental health-sensitive approaches is essential. Schools that adopt inclusive policies not only increase learning opportunities but also create supportive environments that lower stigma, strengthen resilience, and foster psychosocial well-being^[^23,24^]^. Addressing this nexus is important for improving the mental health and academic outcomes of students with learning disabilities in SSA.

## Methodology

This is a narrative review designed to consolidate findings on the relationship between mental health and educational support strategies for students with learning disabilities in SSA. A narrative review approach was considered the most appropriate due to the broad scope of the topic and the heterogeneity in study designs, such as epidemiological surveys and qualitative studies, as well as multiple sources, including organizational reports, NGO-led program evaluations, and national policy documents. This approach allows for an interpretive and contextual synthesis of evidence rather than generating combined results, which is essential for understanding educational and mental health systems in SSA, where relevant insights are often overlooked in reviews with strict inclusion rules^[^25–27^]^.

### Search strategy

A literature search was conducted using databases such as PubMed/Medline, Scopus, and Google Scholar. Search terms involved combining keywords using Boolean operators (AND/OR) such as: “learning disabilities” OR “learning disorders” OR “neurodevelopmental disorders”; AND “mental health” OR “psychosocial” OR “child development”; AND “education” OR “school-based” OR “inclusive education”; AND “Sub-Saharan Africa” OR specific SSA country names. Gray literature was retrieved from the United Nations Children’s Fund (UNICEF), United Nations Educational, Scientific and Cultural Organization, and WHO repositories, alongside Non-Governmental Organization project reports. Manual hand-searching of reference lists and Google searches was also conducted to capture important policy documents^[^27,28^]^.

#### Inclusion and exclusion criteria

Several study types, including empirical studies (qualitative, quantitative, and mixed methods) published between 2010 and 2025, were included, as well as systematic reviews, policy analyses, and program evaluations addressing learning disabilities and mental health outcomes in school-aged children and adolescents in SSA. Studies conducted outside the region, opinion pieces without an empirical or analytical basis, and papers that focused solely on clinical interventions without relevant educational or psychosocial components were excluded, as the review focused on interventions applicable within educational contexts. Additionally, non-English language studies were excluded, which may introduce language bias and limit the representation of relevant insights from non-Anglophone regions.

##### Data extraction and synthesis

Data extracted from included studies comprised study context, population, design, identification tools, intervention components, outcomes, and reported barriers or facilitators. Due to the heterogeneity of these studies, findings were thematically synthesized under four domains: (1) mental health challenges, (2) educational support strategies, (3) barriers to implementation, and (4) policy and practice opportunities. Best-practice guidance for transparency in narrative reviews^[^27,29^]^ was followed to ensure rigor and reproducibility.

Recent literature by Guo *et al*^[^30^]^ demonstrates the rapid growth of AI tools such as ChatGPT in medical research. However, concerns related to inaccuracy, bias, and transparency issues highlight the need for researchers to clearly report the use of AI in research. This manuscript adheres to the TITAN 2025 guidelines for transparent reporting of the use of AI in scientific writing^[^31^]^. An AI-assisted tool was used during the manuscript revision phase to check for grammatical clarity and enhance readability. This tool was not used for study design, data extraction, data synthesis, or the interpretation of findings. All interpretations and conclusions in the review remain solely those of the authors.

## Thematic findings

### Mental health challenges among students with learning disabilities

This section explores the mental health challenges faced by students with learning disabilities in SSA. Although the focus is on school-aged children and adolescents, some studies on university students are included as they provide insight into the persistence of mental health challenges associated with learning disabilities across different educational levels. Adeniyi and Adeniyi^[^32^]^ and Franz *et al*^[^33^]^ highlighted that students with learning disabilities have an increased rate of depression, anxiety, substance use, mood disturbances, and social stigma.

A longitudinal study of university students with ADHD in South Africa by Adeniyi and Adeniyi^[^32^]^ found that these students experience depression, anxiety, and substance use due to the comorbidity of ADHD with other mental disorders. Similarly, Franz *et al*^[^33^]^ reported that adolescents with autism, because of a lack of emotional stability, tend to have mood swings, including moodiness and irritation, which lead to stigmatization from others, impacting their social lives and sometimes resulting in depression. This shows that students with learning disabilities across various educational levels face mental health challenges regardless of their age.

In contrast, Aydin *et al*^[^34^]^ in Ethiopia did not focus on clinically diagnosed learning disabilities but highlighted that students with perceived learning difficulties are also at risk of depression and substance use. The cross-sectional study was conducted using the Patient Health Questionnaire (PHQ-9) to assess depressive symptoms and substance use among university students facing learning difficulties. Results showed that 24.6% of students used substances, with alcohol being the most common. Additionally, 71.4% of students exhibited depressive symptoms, including poor memory, low attention span, disrupted sleep patterns, loss of interest in daily activities, and suicidal thoughts, with loss of interest being the most common symptom, leading to poor academic performance. This suggests that both diagnosed and perceived learning challenges can increase the risk of depression and substance use among students.

The limitations are that the study in Ethiopia was conducted when students were close to exam season; thus, stress and exam phobia may have increased the results of depressive symptoms in the university students. Additionally, recruiting parents of adolescents with autism as participants for the study in Ghana, as opposed to conducting direct interviews with the students, may have also affected the complete accuracy of the results obtained.

With the mental health challenges identified, there is a need to investigate the support available for these students.

#### Educational and mental health support strategies in practice

This section addresses available educational and mental support strategies in practice for students with disabilities in SSA. One of the ways to support students with learning disabilities is by developing centers made specifically to address their needs, as reported by Adeniyi and Adeniyi^[^32^]^.

In 2016, the Center for Early Development, Learning and Care was established in Ibadan, Nigeria, to support children with disabilities such as autism, ADHD, and other intellectual disabilities^[^31^]^. The services offered by the center include assessments such as structured and unstructured interviews, checklists, intelligence, and rating scales for evaluating Intelligence Quotient, depression rates, ADHD, and Autism in children. They also provide special education and various therapies, including Speech and Language Therapy, Sensory Integration Therapy, Occupational Therapy, Play Therapy, and Behavioral Analysis. This center has assessed over 500 clients since its establishment. Despite the importance of this center, it lacks qualified professionals due to the insufficient number of special education specialists in Nigeria.

Prior research by Franz *et al*^[^33^]^, Aydin *et al*^[^34^]^, and Washington-Nortey *et al*^[^35^]^ has identified interventions that serve both children with disabilities, as well as their caregivers and teachers, across Nigeria, South Africa, Kenya, and Ethiopia.

The use of the Picture Exchange Communication System – an alternative communication method for children with autism – as well as operant conditioning to teach sounds and words, has been reported among teachers in Nigeria, South Africa, and Kenya. Medications such as antipsychotics, stimulants, and antidepressants were also used to manage students’ behavioral challenges^[^33^]^. Since disabilities impair children’s ability to be independent, they need to be taught self-management strategies such as self-evaluation, goal setting, self-charting, and self-monitoring to enable them to function and carry out daily activities effectively^[^34^]^. Using baseline, reversal, and alternating treatment study designs, self-monitoring was the most commonly used self-management strategy amongst the children^[^34^]^.

Furthermore, Washington-Nortey *et al*^[^35^]^ used a cluster randomized controlled trial to pilot test the World Health Organization’s Caregiver Skills Training intervention in Kenya and Ethiopia – an intervention that trains caregivers with the skills and knowledge needed to facilitate learning for children with disabilities. Results from the test showed that the caregivers reported the feasibility and effectiveness of the intervention, as it helped reduce the children’s challenges. This indicates that caregivers and teachers are important elements in administering interventions to support children with disabilities. However, a limiting factor to the overall effectiveness of these interventions is that they have only been tested on children with autism; thus, no evidence-based information has been provided to show their effectiveness on children with other types of disabilities.

##### Barriers to implementation in SSA

This section analyzes the barriers that exist in implementing mental health and educational support strategies for students with learning disabilities in SSA. Saade *et al*^[^36^]^ and Chirowamhangu^[^37^]^ identified multiple barriers across Ethiopia, South Africa, and Nigeria.

More specifically, three barriers were identified among parents of children with disabilities and mental health conditions^[^36^]^. The first is the preference for traditional treatments and practices, some of which are harmful, such as self-harm and sacrificing animals, among other practices. The second barrier is stigmatization from others, which makes parents ashamed of their children’s condition and leads them to keep it secret, ultimately resulting in not seeking help. The third barrier is a lack of mental health literacy, as parents are unfamiliar with the various mental health conditions faced by their children and the management strategies available. This evidence points to the need to address mental health literacy at the community level to train parents and caregivers to understand mental health and disabilities and to curb the stigma.

These barriers can also be categorized into government and school^[^36^]^. At the government level, limited priority is given to inclusive education, as seen in South Africa, where inclusive education policies exist but are not implemented. At the school level, there is an insufficient number of special educators and proper teaching aids for students with disabilities. There is also the stigma that makes mainstream schools reject the enrolment of students with disabilities, and those who eventually get in suffer bullying from peers and teachers.

Relatedly, there are limited special needs schools for children with disabilities and insufficient psychosocial experts to help identify learning challenges^[^37^]^. This suggests that the government pays little attention to inclusive education; thus, there is a need to develop and implement better policies and establish more special needs schools, furnished with adequate resources and qualified experts.

A key gap in these studies is the uncertainty about whether the researchers assessed the mental health symptoms of the children with disabilities to confirm their conditions before investigating barriers to the implementation of the strategies.

##### Opportunities and gaps for policy and practice

Having studied the various interventions and barriers to implementation, this section examines existing gaps, tools, and strategies that can be effective, while highlighting growth opportunities. Several studies, including those by Neille and Penn^[^38^]^ and Clark *et al*^[^39^]^, have documented gaps in policy implementation and the limited availability of research-based information on the experiences of people with disabilities in rural contexts.

Policies and models of interventions have been established in South Africa to protect the rights of persons with disabilities^[^38^]^. However, the challenges in rural settings, such as discrimination, social exclusion, barriers to accessing services, and lack of transparency in the implementation of government policies, result in only a limited number of persons in that area benefiting from the policies. These barriers are not only physical but also sociocultural and sociopolitical in nature. Two key strategies have been highlighted to address these implementation gaps, which include the use of culturally sensitive approaches, such as narrative inquiry to explore the lived experiences of people with disabilities in rural areas, and the replacement of the one-size-fits-all approach with context-specific policies, such as inclusive ECE supported by culturally appropriate tools^[^38^]^.

Similarly, inclusive ECE is poorly implemented in some SSA countries like South Africa and Kenya^[^39^]^, largely due to misconceptions within the community that children with disabilities cannot be educated, and the belief that the responsibility of implementing inclusive ECE rests solely on the national government, thereby reducing state-level engagements. Strengthening accountability and transparency at the governance levels is, therefore, essential for inclusive ECE to be prioritized and effectively implemented.

Several strategies in practice, as indicated by Okyere *et al*^[^40^]^, Carew *et al*^[^41^]^, and Hunt *et al*^[^42^]^, have been identified to support students with learning disabilities across South Africa, Ghana, Kenya, and Botswana. These practices are categorized into the biosystemic level (child-related factors such as peer support), microsystemic level (teacher-related factors such as teachers’ knowledge and skills), mesosystemic level (teacher-parent collaboration), and macrosystemic level (environmental factors)^[^40^]^. In Kenya, an NGO-led inclusive education intervention was reported to enhance students’ learning outcomes^[^41^]^. To ensure sustainability, the intervention adopts a holistic approach, including projects such as raising awareness about disabilities among caregivers and communities to challenge stigma, peer support activities, training teachers on inclusive education and disability rights, and advocating for policy change at the county and national levels. Additional interventions in practice include educational attainment support and accessible learning environments^[^42^]^.

Overall, the literature indicates that gaps exist in government transparency, policy implementation, and the application of inclusive ECE and culturally sensitive tools. However, in contexts where national implementation of interventions is limited, NGO-led inclusive education interventions can alternatively be employed for support.

## Discussion

### Synthesis of themes

The literature presents a clear and urgent challenge: a high prevalence of mental health problems exists among adolescents in SSA^[^43^]^, a rate that is at least doubled for children with neurodevelopmental disorders such as intellectual disability^[^44^]^. This mental health burden is exacerbated by significant educational exclusion, with many children and adolescents living with disabilities in the region remaining out of school^[^45^]^.

Schools are consistently identified as strategic and feasible platforms for delivering vital Mental Health and Psychosocial Support (MHPSS), particularly in low- and middle-income countries (LMICs) where access to specialist care is profoundly limited^[^23,44^]^. However, a substantial gap exists between this potential and the current reality. The implementation of inclusive education and school-based MHPSS is hindered by systemic barriers, including insufficient teacher training, a lack of resources, pervasive stigma, and ambiguous government policies^[^5,23,45^]^. While studied interventions show promise for improving outcomes like social skills, their generalizability is constrained by methodological weaknesses and a predominant focus on special education rather than mainstream, inclusive environments^[^23^]^.

#### Comparison with global evidence

The heightened vulnerability of children with intellectual disabilities to mental health problems is a global phenomenon, with prevalence rates consistently doubling those of their typically developing peers in both high-income countries (HICs) and LMICs^[^44^]^. However, the overall adolescent mental health burden in SSA appears high compared to rates in HICs^[^43^]^. A significant divergence appears in the strategic approach to intervention. HICs often employ a structured, three-tiered public health model (universal, selective, and indicated) for school-based mental health, creating a continuum of care^[^46^]^. This systematic framework is less apparent in the LMIC context, where interventions are more commonly standalone, multimodal approaches, or educational workshops^[^23^]^.

Implementation challenges are universal, but their nature differs starkly. In HICs, the focus is often on improving the fidelity of evidence-based practices and navigating the complexities of integrating health and education systems^[^46^]^. In contrast, SSA faces more foundational barriers, including a severe lack of basic infrastructure, teacher training, and resources to manage large, overcrowded classrooms^[^5,45^]^.

##### Implications for education and mental health practice

The evidence points to the need to invest in and empower teachers. This requires moving beyond one-off workshops to provide sustained, practical training and support in inclusive pedagogy, as well as the skills to identify and manage common mental health challenges^[^5,23,45^]^.

At a systemic level, a principle of proportionate universalism is essential, whereby universal mental health promotion programs are designed to be inclusive of all learners while providing more intensive, targeted support for those with greater needs, such as children and adolescents living with disabilities^[^44^]^. This integrated approach, which combines classroom-level environmental support with individual student interventions, has shown promise in HICs and could be adapted to LMICs^[^46^]^. Finally, governments must create and implement clear, well-funded policies for inclusive education^[^5,45^]^.

##### Limitations

This review has several limitations that should be considered when interpreting the findings. It adopts a narrative review approach, which helps to synthesize diverse evidence; however, it does not involve a systematic study selection process or an assessment of study quality. This may lead to selection bias, which limits the reproducibility of the findings. Additionally, the included studies, being heterogeneous in design, make it difficult to compare and combine findings to reach a statistical result.

Furthermore, including gray literature, such as organizational reports, although providing contextual insights, may affect the strength of the evidence due to the absence of rigorous peer review. The exclusion of non-English studies may also introduce language bias, including the underrepresentation of relevant findings from non-Anglophone regions. Variation in the interpretation of findings may equally arise due to the additional review of a small number of studies focusing on university students. Lastly, the concentration of included studies on a few SSA countries may lead to the uneven representation of evidence across the region.

## Conclusion

The prevalence of childhood disabilities in Africa is increasing, making early identification and intervention an urgent public health and education concern. This necessitates refocusing attention on multifaceted and integrative strategies that combine health, education, and community-based support systems to mitigate the prevalence and effects of developmental impairments and promote long-term inclusion. Given the limited formal support systems available, parents, caregivers, and healthcare professionals play a critical role in the identification, evaluation, and care of children and adolescents with disabilities. Enhancing their involvement improves early access to support and increases educational and mental health outcomes. This ensures that children and adolescents with learning disabilities not only have access to education but are also supported and able to thrive within society.

### Recommendation

Grounded in the Social Model of Disability, which focuses on restructuring society to be more inclusive by eliminating systemic and environmental barriers, the following recommendations are proposed to improve access, participation, and learning outcomes for students with learning disabilities in SSA:

#### Improving access to early support and education

The use of locally created and validated assessment tools, alongside the involvement of parents and caregivers in the evaluation of students with learning disabilities, would support early identification and proper placement of students in suitable educational environments.

##### Enhancing participation in inclusive educational settings

The adoption of a collaborative approach involving families, teachers, special educators, and therapists can strengthen the active participation of students with learning disabilities in classroom and school activities. Embedding therapeutic support within educational environments can further support students’ learning processes.

Additionally, raising public awareness of learning disabilities will help reduce stigma, which is a major barrier to participation in educational environments.

##### Improving learning outcomes and system-level support

Strengthening the development and implementation of inclusive education policies can improve learning outcomes for students with learning disabilities. Additionally, addressing communities’ sociocultural perceptions of disability can support the effectiveness of these policies.

Similarly, the adoption and implementation of the Convention on the Rights of Persons with Disabilities by African governments can enhance accountability, protect the rights of persons with disabilities, and create more effective, inclusive education systems.

## Data Availability

All datasets generated and/or analyzed in this study are available in the published article and its supplementary information files.

## Peer and provenance statement

Not commissioned; externally peer-reviewed.
